# Kinetics of the Reaction of Pyrogallol Red, a Polyphenolic Dye, with Nitrous Acid: Role of ●NO and ●NO_2_

**DOI:** 10.3390/molecules200610582

**Published:** 2015-06-08

**Authors:** Estefania Hugo, Jael Reyes, Elisa Montupil, Raquel Bridi, Eduardo Lissi, Ana Denicola, María Angélica Rubio, Camilo López-Alarcón

**Affiliations:** 1 Facultad de Química y Biología, Universidad de Santiago de Chile, Santiago 8320000, Chile; E-Mails: estefahugo@gmail.com (E.H.); eduardo.lissi@usach.cl (E.L.); maria.rubio@usach.cl (M.A.R.); 2 Departamento de Farmacia, Facultad de Química, Pontificia Universidad Católica de Chile, Santiago 7820436, Chile; E-Mails: jcreyes@uc.cl (J.R.); edmontup@uc.cl (E.M.); rbridi@uc.cl (R.B.); 3 Laboratorio de Fisicoquímica Biológica, Facultad de Ciencias and Center for Free Radical and Biomedical Research, Universidad de la República, Montevideo 11400, Uruguay; E-Mail: denicola@fcien.edu.uy; 4 Cedenna, Universidad de Santiago de Chile, Santiago 8320000, Chile

**Keywords:** kinetics, nitrous acid, nitric oxide, nitrogen dioxide, pyrogallol red, phenols

## Abstract

In the present work we studied the reaction under gastric conditions of pyrogallol red (PGR), a polyphenolic dye, with nitrous acid (HONO). PGR has been used as a model polyphenol due to its strong UV-visible absorption and its high reactivity towards reactive species (radicals and non-radicals, RS). The reaction was followed by UV-visible spectroscopy and high performance liquid chromatography (HPLC). A clear decrease of the PGR absorbance at 465 nm was observed, evidencing an efficient bleaching of PGR by HONO. In the initial stages of the reaction, each HONO molecule nearly consumed 2.6 PGR molecules while, at long reaction times, *ca.* 7.0 dye molecules were consumed per each reacted HONO. This result is interpreted in terms of HONO recycling. During the PGR-HONO reaction, nitric oxide was generated in the micromolar range. In addition, the rate of PGR consumption induced by HONO was almost totally abated by argon bubbling, emphasising the role that critical volatile intermediates, such as •NO and/or nitrogen dioxide (•NO_2_), play in the bleaching of this phenolic compound.

## 1. Introduction

The reaction of phenolic compounds with nitrous acid (HONO) is a matter of current interest due to its occurrence in the gastrointestinal track [1,2,3]. This reaction can be relevant since phenols included in the human diet could decrease HONO concentration in the gastric lumen. Taking into account the well-known capacity of HONO to react with amines forming carcinogenic nitrosamine derivatives, this reaction could also be associated with a decrease of the deleterious effect caused by high concentrations of this reactive species in the gastric cavity [4,5].

In addition to the latter, in the last decades the beneficial effects on the human health related to the HONO-phenol reaction have been emphasised by the discovery that nitric oxide (•NO) is generated as a product. In fact, it has been demonstrated, *in vitro* and *in vivo*, that the interaction of phenolic derivatives contained in foods and beverages with HONO leads to the release of •NO in the micromolar range [2,3,6,7,8]. This process has been associated with a possible protection of the gastric mucosa by phenols and has been highlighted as a novel beneficial effect on the human health produced by a phenol-rich diet [2].

The release of •NO during the reaction of HONO with phenols has been explained by their capacity to reduce HONO, by an one electron mechanism in according to Reaction (1) [2]:

HONO + Phenol-H → Phenol• + •NO + H_2_O (1)

However, HONO in aqueous solutions is in equilibrium with different reactive nitrogen species (N_2_O_3_, •NO, •NO_2_) that also could be relevant in the •NO release process. Due to the complexity of the system, which includes the production of reactive gaseous intermediates and different reaction pathways, currently there are many unanswered questions regarding the mechanism underlying the reactions of phenolic compounds with HONO [9].

Pyrogallol red (PGR) is an *ortho*-diphenolic dye with a main chemistry similar to that of other polyphenols present in the human diet. In fact, PGR reacts efficiently with reactive oxygen (ROS) and nitrogen (RNS) species, such as peroxyl radicals, peroxynitrite, hypochlorite and superoxide anion radical [10,11,12,13,14]. The consumption of PGR, usually complete at relatively short times, can be easily followed by absorption spectroscopy in a wavelength range of minimal interferences. A characteristic of this compound is the reactivity of its primary phenolxyl radical, a derivative prone to release a second hydrogen atom to produce stable quinone-like oxidation products [15].

Considering the characteristics of PGR as a model molecule of *ortho*-diphenols and the relevance of the oxidative processes mediated by HONO in the gastrointestinal track, in the present work we studied the interaction of PGR with HONO under gastric conditions. We present kinetic data regarding such reactions with emphasis on the generation of •NO.

## 2. Results and Discussion

### 2.1. Studies of the PGR-HONO Reaction

#### 2.1.1. Kinetics of PGR Removal by HONO

**Figure 1:**
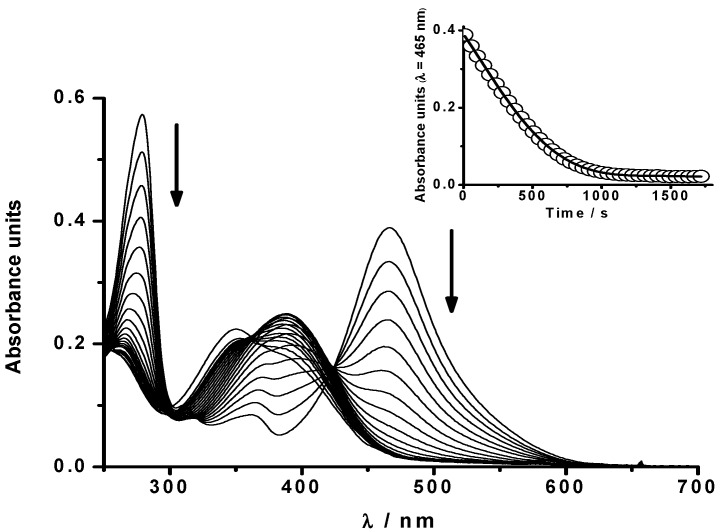
Bleaching of PGR (20 µM) induced by HONO 40 µM between 0 and 50 min of reaction in SGM at 37 °C. Inset: Time course of the reaction followed at 465 nm.

As Figure 1 shows, the addition of HONO to a PGR solution readily bleached its absorbance at 465 nm and 280 nm together with the appearance of new bands in the 300–430 nm region. The lack of a clear isosbestic point indicates the presence of reactive intermediates, particularly at long reaction times (see below). Also, the fact that the PGR absorbance at 465 nm reaches very low values (inset of Figure 1) indicates that light absorption by the products at this wavelength can be considered as negligible.

**Figure 2:**
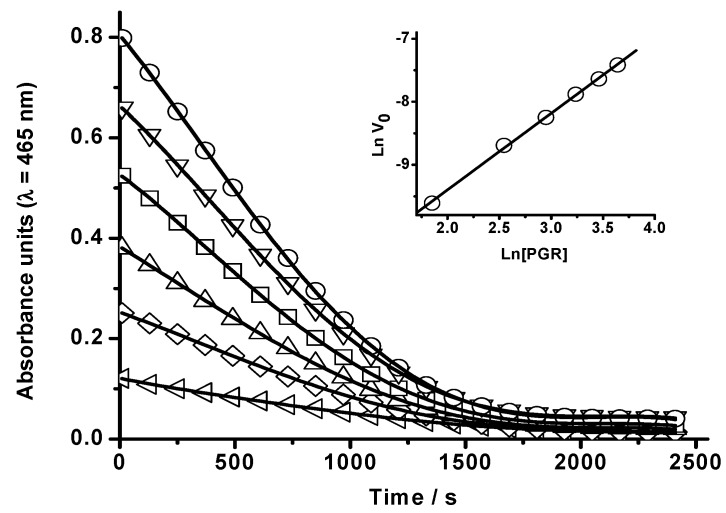
Influence of the PGR concentration on its HONO-mediated bleaching. [PGR] = 6 μM (◁), 13 μM (◊), 20 µM (Δ), 26 μM (□), 32 μM (▽), and 38 μM (○). [HONO] = 20 µM. The reaction was followed at 465 nm in SGM at 37 °C. Inset: Ln(initial reaction rate, V_0_) *vs.* Ln[PGR].

**Figure 3:**
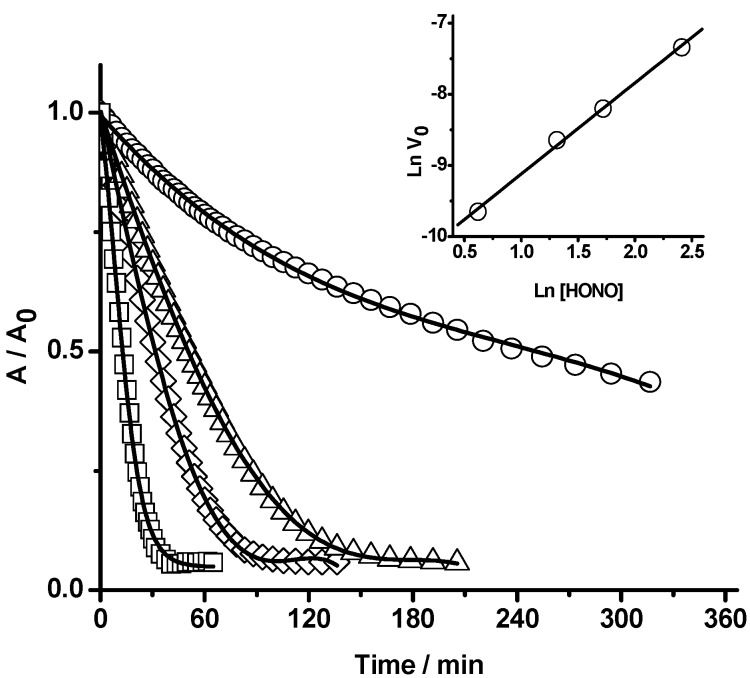
Influence of HONO concentration on the PGR bleaching. [HONO] = 2 μM (○), 3 μM (Δ), 6 μM (◊), and 11 μM (□). [PGR] = 22 µM. The reaction was followed at 465 nm in SGM at 37 °C. Inset: Ln(initial reaction rate, V_0_) *vs.* Ln[HONO]^0^.

From values of the initial reaction rate, obtained at a fixed HONO concentration and over a range of PGR concentrations (from 6.4 to 38 µM), the PGR reaction order was estimated. Typical results, obtained at 37 °C, are presented in Figure 2. As it is shown in the inset of this figure, the Ln(initial reaction rate, V_0_) *vs*. Ln[PGR] plot showed a linear behaviour with a slope of 1.2 ± 0.1. In addition, initial reaction rates at a fixed PGR concentration, but in the presence of different HONO concentrations (from 1.9 to 11 µM) were estimated (Figure 3). From the Ln(initial reaction rate, V_0_) *vs*. Ln[HONO]^0^ plot (inset of Figure 3) was estimated a reaction order in HONO equal to 1.3 ± 0.2. These reaction orders (of PGR and HONO) are somehow higher than those obtained by Vione *et al.* [16] who reported that the initial rates of phenol nitration by HONO were nearly proportional to both reactant concentrations.

**Figure 4:**
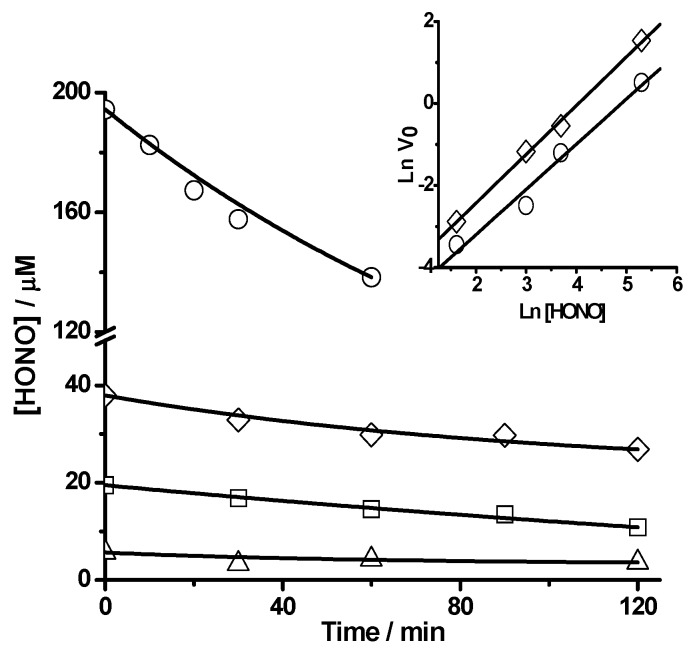
Consumption of HONO during the PGR-HONO reaction. PGR (20 µM) was incubated with HONO at 5 µM (Δ), 20 µM (□), 40 µM (◊), and 200 µM (○) in SGM. The HONO concentration was determined according to Griess assay. Temperature = 25 °C. Inset: Ln(initial reaction rate, V_0_) *vs.* Ln[HONO]^0^ plot. V_0_ was measured by the consumption of PGR (◊) and HONO (○).

Additional support of the reaction between PGR and HONO can be obtained following the consumption of HONO. As presented in Figure 4, the concentration of HONO (measured by Griess assay) decayed during its incubation in the presence of PGR at 25 °C in SGM. As it is shown in the inset of this figure, the Ln(initial reaction rate, V_0_) *vs.* Ln[HONO]^0^ plot showed a linear behaviour with a HONO reaction order of 1.1 ± 0.1. The inset of Figure 4 also includes initial rates values obtained in the same experimental conditions but measured by PGR absorbance decrease. These values gave a similar slope (HONO reaction order of 1.18 ± 0.04), however, showed that the rate of PGR consumption was faster than the rate of HONO consumption. In fact, a comparison of both sets of data indicate that:

Rate_PGR_ ≈ 2.6 Rate_HONO_ (2)

implying that, at the early stages of the process, *ca.* 2.6 molecules of PGR are consumed per each molecule of HONO over all the concentration range considered. Nonetheless, at high conversions, the number of PGR molecules consumed per each HONO molecule was considerably larger. In fact, the data given in Figure 3 show that 3 µM of HONO is enough to bleach totally the absorption of 22 µM PGR. This indicates that each HONO molecule is able to remove at least seven PGR molecules. This was further confirmed by results obtained at 25 °C in the dark where, after 24 h of reaction, a total bleaching of PGR (20 µM) promoted by HONO 3 µM was observed (data not shown). These results could be explained by short chain reactions in the PGR removal and/or by the presence of a HONO recycling mechanism (*vide infra*).

**Figure 5:**
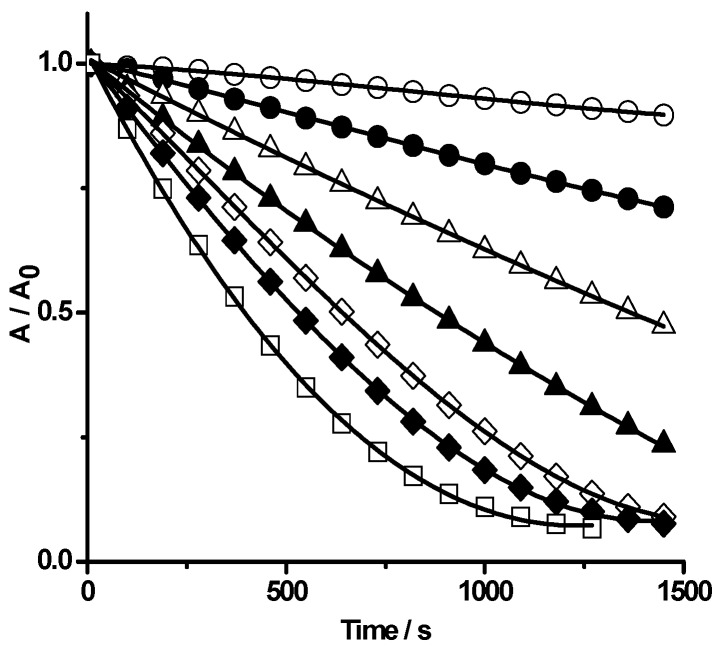
Influence of pH on the PGR (20 µM) bleaching mediated by HONO (40 µM). The time course of the reaction was followed by UV-visible spectroscopy (λ = 465 nm) at different pH: 1.7 (□), 2.0 (♦), 2.2 (◊), 2.4 (▲), 2.7 (Δ), 3.0 (●), and 3.4 (○).

To further characterize the reaction of PGR with HONO, we studied the effect of pH and temperature on the initial reaction rate. As Figure 5 shows, the increase of the solution pH from 1.6 to 3.4 significantly reduced the rate of the process. This dependence would imply that the protonated acid (HONO) is the reactive species (pKa of HONO *ca.* 3.5). This conclusion is in agreement with previously reported data regarding the rate of phenol oxidation mediated by HONO in a similar pH range [17]. The rate of the process, measured by the consumption of PGR, increased with the temperature of the media. The observed differences in the rates between 25 and 37 °C correspond to an activation energy of *ca*. 12 kcal/mol. If it is assumed that the overall rate follows a second order kinetics, the pre-exponential factor is *ca*. 10^11^ M^−1^·s^−1^. These values are compatible with those expected for a process whose rate determining step is a simple bimolecular process [16].

#### 2.1.2 Nitric Oxide (•NO) Formation

**Figure 6:**
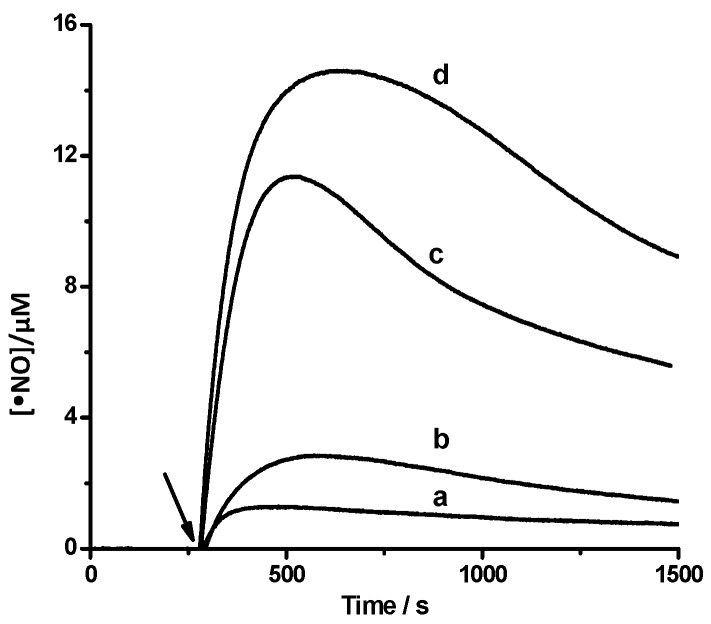
Production of •NO during the reaction of PGR with HONO. Time course of the •NO production upon addition of 100 µM HONO (arrow) in the absence (*line a*) and presence of PGR at final concentrations; 5 µM (*line b*); 30 µM (*line c*); 50 µM (*line d*). The solutions were incubated in SGM at 25 °C.

The reaction of PGR with HONO produces, as others phenolic compounds [2,6], significant amounts of •NO. Figure 6 shows the formation of •NO during the reaction of PGR (5–50 µM) and HONO (100 µM). Remarkable aspects of these data are the large initial slope of the plots and the •NO consumption that takes place at long reaction times. At short reaction times •NO production takes place at a rate faster than that of PGR consumption. However, this rather surprising fact results from the different experimental settings under which •NO production (absence of gas phase) and PGR consumption (presence of gas phase) were measured. In fact, if the rate of PGR consumption is corrected by the •NO present in the gas phase (a factor of *ca.* 3.0) the rate of both processes becomes similar.

#### 2.1.3. Studies by HPLC-DAD Technique

**Figure 7:**
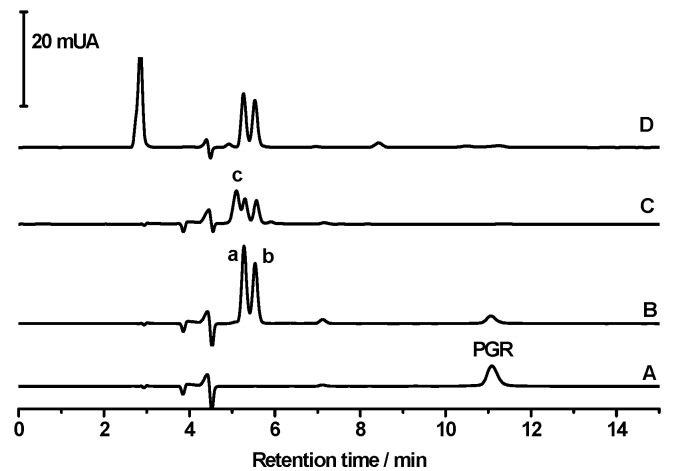
Time course of the reaction of PGR with HONO or peroxyl radicals followed by HPLC. PGR (20 µM) was incubated with HONO (300 µM) in SGM. Chromatograms were obtained (λ = 380 nm) at different incubation times: A: 0 min; B: 10 min. and C: 110 min. D: Chromatogram obtained after 30 min of the PGR (20 µM) incubation with AAPH (10 mM) in phosphate buffer (pH 7.4) at 37 °C.

**Figure 8:**
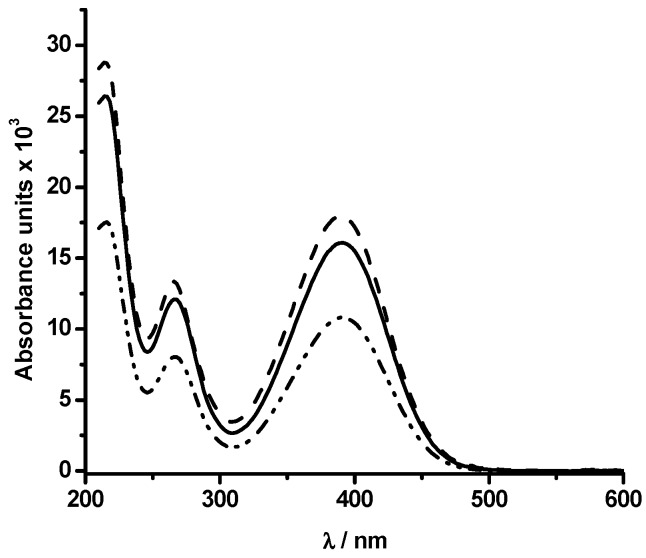
UV-visible spectra of the HPLC peaks at 5.6 min (dash line) and 5.1 (solid line) obtained during the reaction of PGR (20 µM) and HONO (300 µM). Dash-dot-dot line: UV-visible spectrum of the HPLC peak at 5.6 min obtained from the reaction of PGR (20 µM) and AAPH-derived peroxyl radicals.

To gain further insights into the reaction of PGR with HONO, the formation of products was studied by HPLC-DAD. As Figure 7 shows, the area under the curve of the chromatographic peak of PGR, registered at 11 min, decreased significantly in the presence of HONO (chromatograms A, B and C). The kinetic profiles showed a similar behaviour than those obtained by UV-visible spectroscopy (data not shown). At early stages of the reaction, two products, at retention times of 5.3 and 5.6 min, were registered (peaks a and b in Figure 7). Interestingly, the chromatogram obtained in the first min of the PGR-HONO reaction was similar to the HPLC chromatogram of the PGR- •NO_2_ reaction [15]. These products showed similar UV-visible spectra to those obtained by UV-visible spectroscopy in the first min of the reaction. Nonetheless, at long reaction times or under excess HONO conditions, a third peak, at 5.1 min was registered (peak c in Figure 7). The formation of the latter was related to a decrease of the area under the curve of peaks a and b (Figure 7). Interestingly, peaks a and b were also registered during the PGR oxidation induced by peroxyl radicals (chromatogram D in Figure 7). In fact, peaks a and b obtained either by the reaction of PGR with HONO, •NO_2_ or peroxyl radicals presented similar UV-visible spectra (Figure 8), and were identified as isomers of a PGR-quinone derivative [15].

### 2.2. Reaction Mechanism

In agreement with the studies of the wine-dependent reduction of nitrite to form •NO in stomach conditions [2], the above presented data are compatible with an initial (rate determining) step represented by Reaction (3):

HONO + PGR → PGR• + •NO + H_2_O (3)

Nonetheless, the consumption of PGR mediated by HONO and the related •NO release could also be explained by Reactions (4) to (7):

2HONO ⇄ N_2_O_3_ + H_2_O (4)

N_2_O_3_ ⇄ •NO + •NO_2_ (5)

•NO_2_ + PGR → •PGR + HONO (6)

•NO_2_ + •PGR → Q + HONO (7)

**Figure 9:**
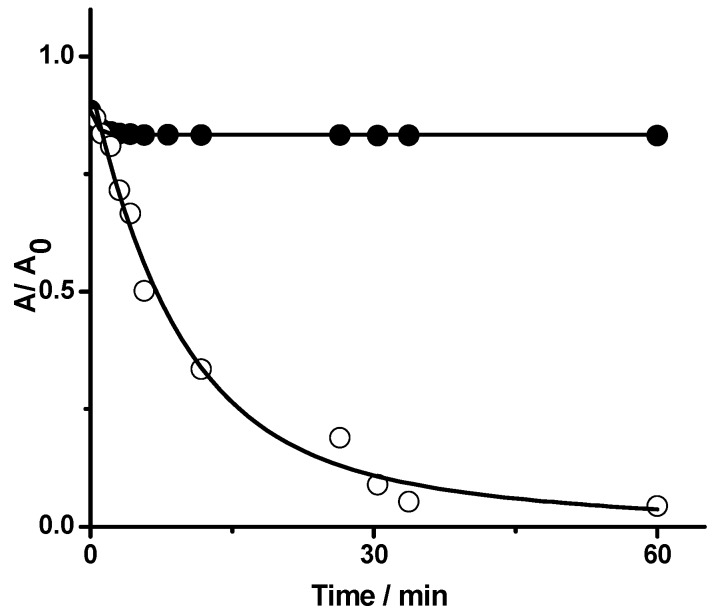
Time-profile of PGR (40 µM) bleaching induced by HONO (100 µM) without (○) and with (●) continuous bubbling of argon. Solutions were incubated in SGM at 25 °C and the consumption of PGR was followed at 465 nm.

The occurrence of Reactions (6) and (7) implies a consumption of PGR and also a release of •NO from the displacement of equilibrium of Reaction (5) to the right. To establish if under our experimental conditions the consumption of PGR is associated with a simple bimolecular reaction with HONO (Reaction (3)) or with •NO_2_, we studied the kinetics of the reaction under a continuous bubbling of argon or oxygen. Interestingly, the consumption of PGR was almost totally inhibited in both conditions (Figure 9). These results could be explained considering that the flux of argon and oxygen removed from the working solution reactive intermediates (•NO and •NO_2_). Moreover, products generated in the first min of the PGR- HONO reaction were similar to products generated in the PGR- •NO_2_ reaction [15]. Taking into account the well-known oxidant property of •NO_2_, (0.99 V at pH 7.0) [17,18] we postulate that the main consumption of PGR in closed systems is due to Reactions (6) and (7).

#### Consumption of PGR by Chain Reactions and/or HONO Recycling Processes

The above mentioned reactions imply an interaction of the PGR radical (PGR^•^) with different RNS derived from HONO. Chain reactions of PGR (that involve PGR^•^) could also be present. These secondary reactions could recycle HONO and/or consume PGR in a chain process. The rather high stoichiometry of PGR consumption, even at low conversions, could be explained in terms of HONO recycling either by Reactions (6) and (7), or by •NO oxidation (Reaction (8)). The •NO_2_ generated in Reaction (8) could react with •NO to form N_2_O_3_ (Reaction (5)) which, after hydration, forms HONO (Reaction (4)).

2•NO + O_2_ → 2•NO_2_ (8)

This sequence of reactions could also account by the •NO consumption observed in the time profiles given in Figure 6. Considering that chain reactions of PGR (that involve PGR•) should generate hydrogen peroxide (H_2_O_2_) as product (Reactions (9) and (10)), we evaluated its formation using Fox methodology [19]. Under our experimental conditions, no significant H_2_O_2_ concentrations (below 1 µM) were detected, arguing against the occurrence of large chain reactions during PGR consumption:

•PGR + O_2_ → Q + •HO_2_ (9)

PGR + •HO_2_ → •PGR + H_2_O_2_ (10)

where Q represents the quinone derivative produced during PGR oxidation.

Reaction (8) is a key step in the oxidation of •NO to •NO_2_ and the subsequent oxidation of phenols (PGR). This reaction is kinetically second order in •NO both in the gas and liquid phases and can take place in the two bulk phases and at the gas/water interface [20]. At room temperature the recommended rate constant is:

k_6_ = 2 × 10^−38^ cm^6^·mol^−2^·s^−1^ (11)

and when the •NO pressure is 1 Torr in air its oxidation amounts to *ca*. 40% in one minute. Under our experimental conditions •NO concentration in the aqueous phase can reach values as high as 10 μM (Figure 6). This implies that, if Henry’s law is considered, the gas phase concentration reaches values of *ca.* 6 Torr, leading to oxidation rates over 40%/min [21]. This high oxidation rate is a lower limit for the total NO oxidation since it calculation disregards the gas phase (and gas liquid interfaces) oxidations. The occurrence of this reaction leads to HONO recycling, according to Reactions (12)–(14).

PGR + HONO → •PGR + •NO + H_2_O (12)

2•NO + O_2_ → 2•NO_2_ (13)

•NO_2_ + •PGR → HONO + Q (14)

and the global reaction represented by Reaction (15).

PGR + 0.5O_2_ → Q + H_2_O (15)

The latter process could explain the large stoichiometric coefficient of PGR, and the role that oxygen could play in the mechanism of PGR consumption elicited by HONO.

## 3. Experimental Section

### 3.1. Chemicals and Solutions

Hydrochloric acid, methanol, ethanol, sodium nitrite and sodium chloride (pa., Merck, Santiago, Chile) were employed as received. Deionized, ultra-pure water from a Easy-Pure II water purification system (Thermo Scientific, Marietta, OH, USA) was employed in all solutions. Experiments were carried out in a NaCl (34 mM) solution (named simulated gastric medium, SGM) adjusted to pH 2.0 with concentrated hydrochloric acid at 25 or 37 °C. Working HONO solutions were prepared from a standard solution (10 mM) made with solid sodium nitrite. The concentration of the standard solution was determined by the Griess methodology [21].

### 3.2. UV-Visible Studies

The consumption of PGR, elicited by the addition of HONO, was evaluated from the decrease in the absorbance intensity measured at 465 nm. Briefly, a reaction mixture containing PGR (4–60 µM) and HONO (5–300 µM) in SGM, was incubated at 25 or 37 °C in the thermostatized cuvette of an 8453 UV-visible spectrophotometer (Agilent, Palo Alto, CA, USA). Cells of 3.5 mL capacity were filled with the working solution leaving a gas-phase volume of 0.5 mL. The reaction was followed till total PGR consumption (in excess of HONO) or to a constant absorbance value (excess of PGR).

### 3.3. Electrochemical Detection of •NO

The release of •NO was followed using an ISO-NO electrode (WPI, Inc., Sarasota, FL, USA) under a nitrogen atmosphere at 25 °C. Solutions of KI-H_2_SO_4_ (0.1 M) plus sodium nitrite (0.7–10 µM) were used to calibrate the electrode. Working solutions containing PGR (5–50 µM) in SGM were incubated in a thermostatized cell. After stabilization of the baseline, a sodium nitrite bolus (100 µM) was added without a measurable gas phase in contact with the working solution. 

### 3.4. High Performance Liquid Chromatography (HPLC) Studies

The time course of the reaction of PGR with HONO was followed by HPLC with a diode array detector (DAD). Chromatograms were obtained using an Agilent 1200 series HPLC system equipped with a Hibar (Merck) RP-18 (5 µm) column (25 cm × 4.6 mm), and a DAD model G1315D. Phosphate (KH_2_PO_4_, 0.1 M, adjusted to pH 2.6 with ortophosphoric acid)/acetonitrile (80/20) was employed as mobile phase. The flow rate was 0.8 mL/min. Solutions of PGR (4–50 µM) were incubated in the absence and presence of HONO (2–300 µM) in SGM. At different times, aliquots were taken and immediately injected into the HPLC system. The reaction was followed at 380 nm. All experiments were performed in duplicate or triplicate.

## 4. Conclusions

HONO promotes the bleaching of PGR involving, in the initial stages, the oxidation of the dye and the release of NO. The NO_2_, generated during the oxidation of the later, is involved in the consumption of PGR. In addition, at long reaction times a HONO recycling mechanism which would explain the high stoichiometry of the reaction is observed. The proposed mechanisms for PGR oxidation could also be extrapolated to the processes that natural polyphenols undergo in the presence of HONO in conditions simulating a gastric environment.
